# Supplementary figures and images for: Transcriptome-wide association study and eQTL colocalization identify potentially causal genes responsible for human bone mineral density GWAS associations
Source: eLife. 2022 Nov 23;11:e77285. doi: 10.7554/eLife.77285 (PMC9683789; doi:10.7554/eLife.77285)

Fig4B Blots

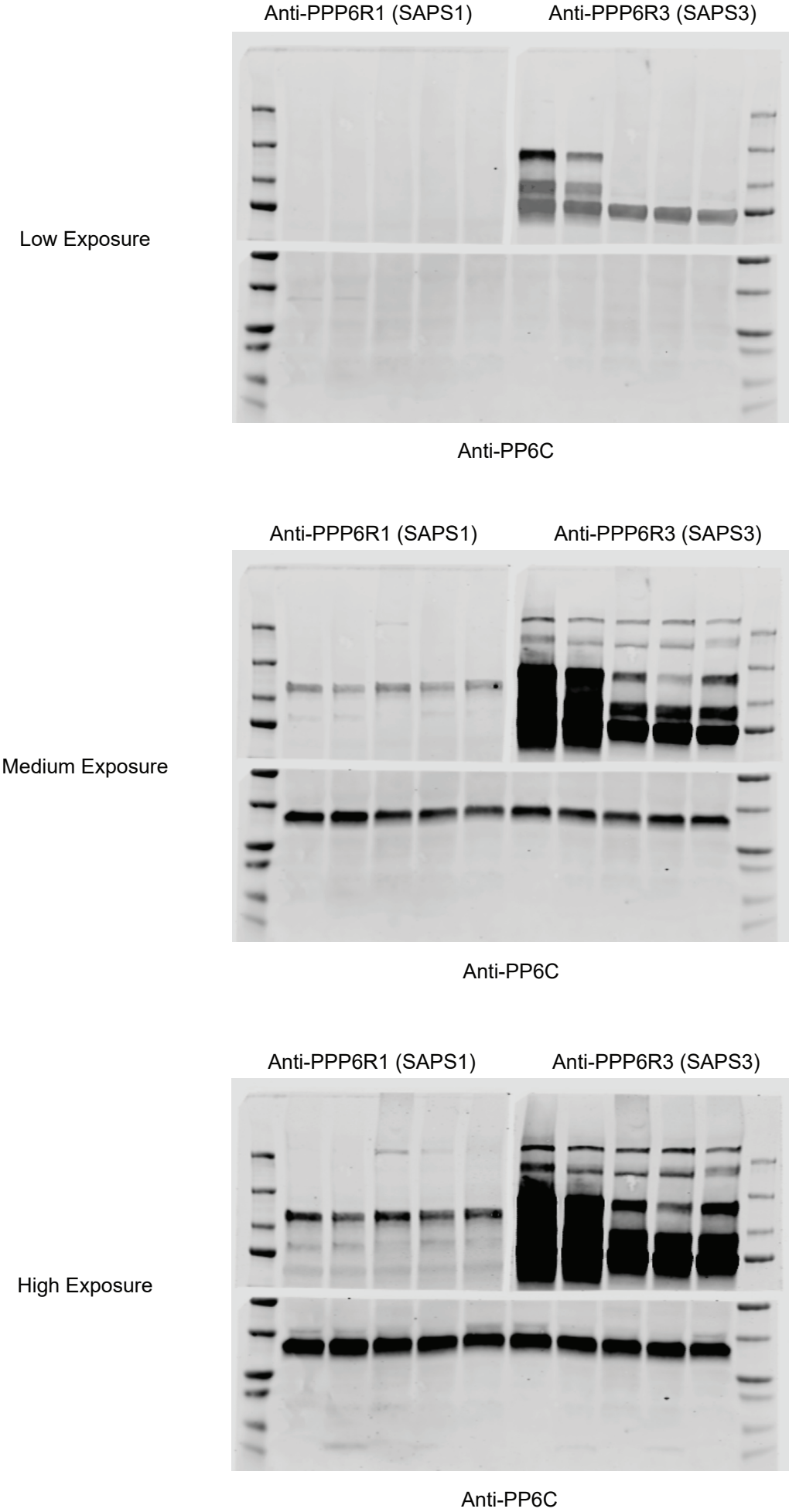

Supplement: Figure 4—source data 1. [file elife-77285-fig4-data1.zip › Figure 4-source data 1.pdf]

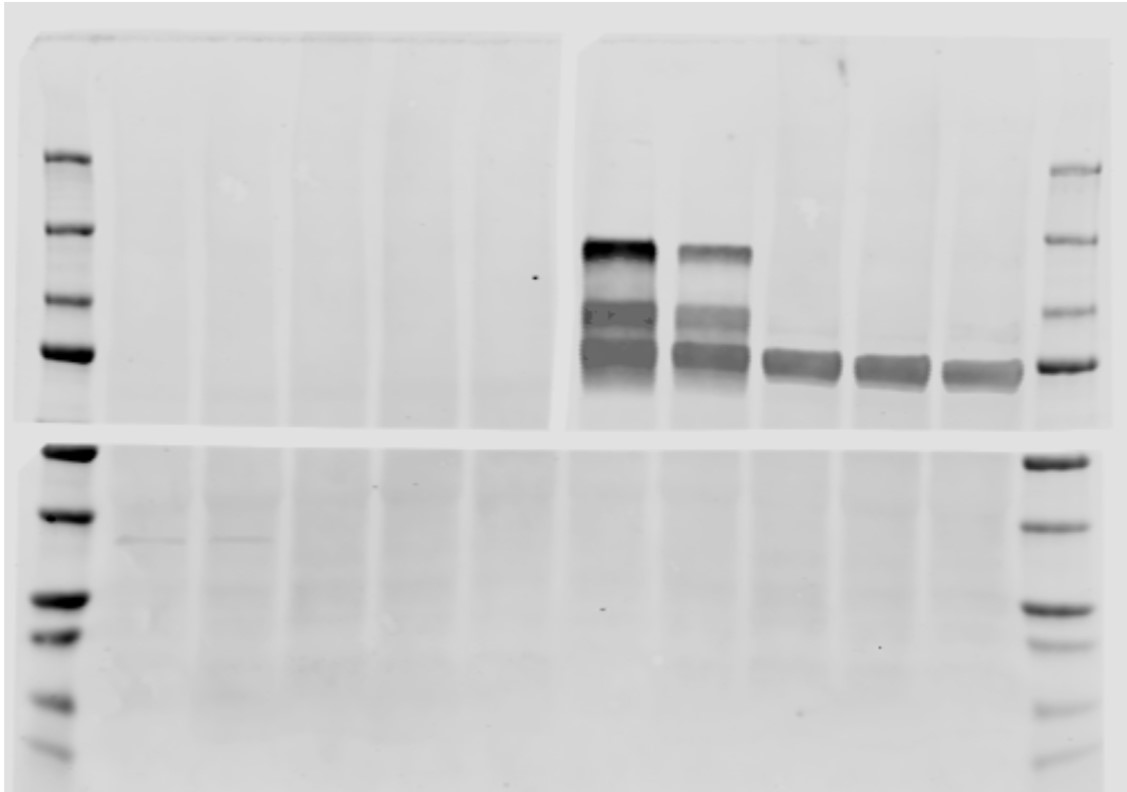

Supplement: Figure 4—source data 1. [file elife-77285-fig4-data1.zip › Figure 4-source data-raw 1.jpg]

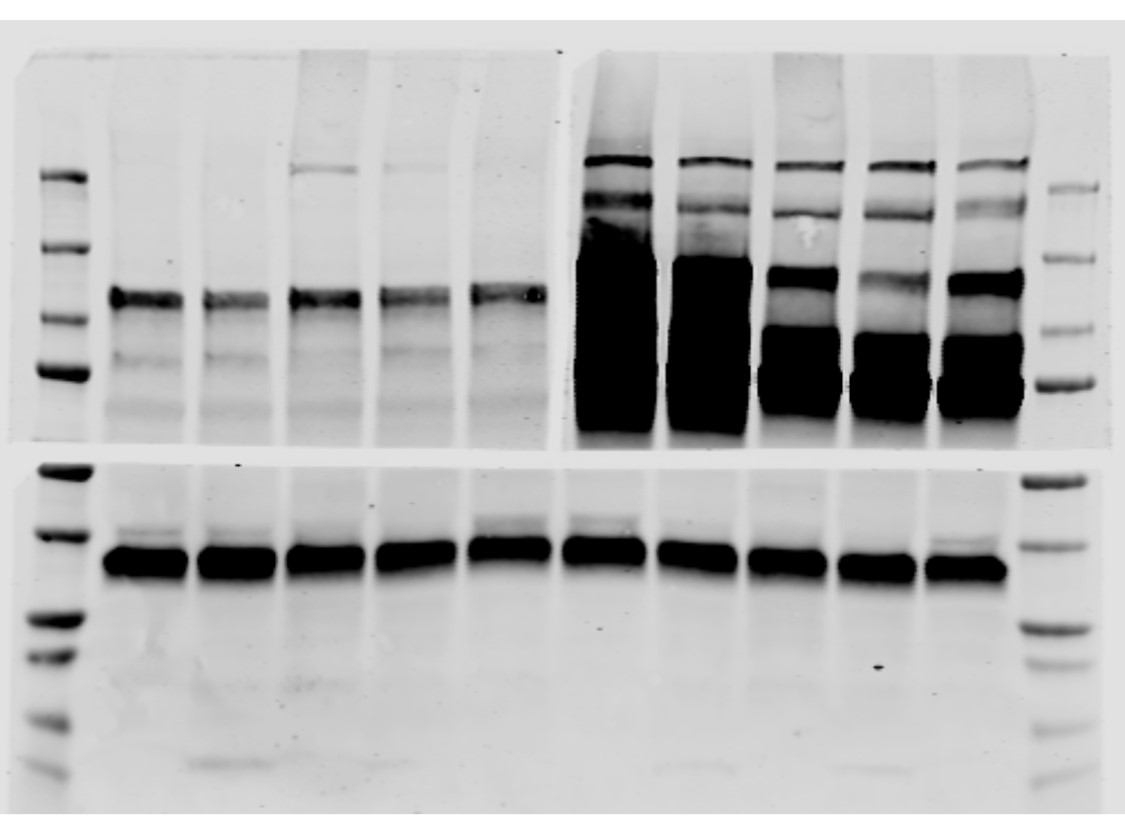

Supplement: Figure 4—source data 1. [file elife-77285-fig4-data1.zip › Figure 4-source data-raw 3.jpg]

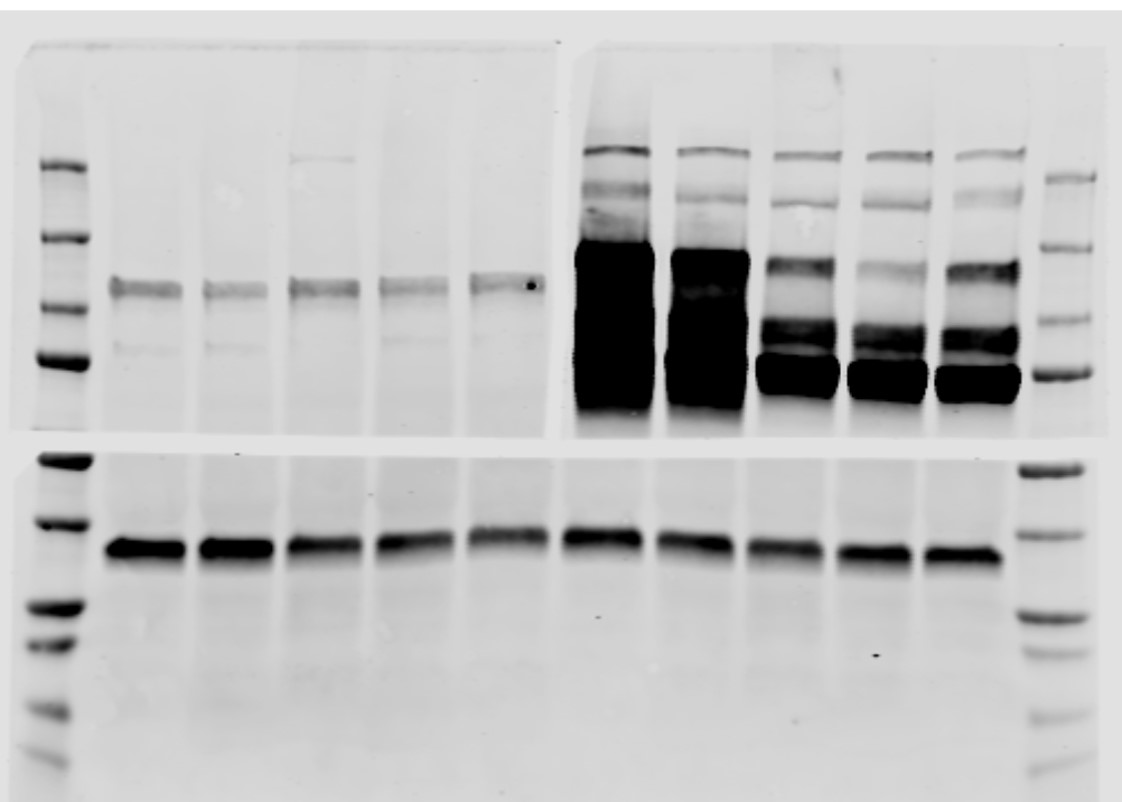

Supplement: Figure 4—source data 1. [file elife-77285-fig4-data1.zip › Figure 4-source data-raw2.jpg]
